# The Legacy of Plant Invasion: Impacts on Soil Nitrification and Management Implications

**DOI:** 10.3390/plants12162980

**Published:** 2023-08-18

**Authors:** Muhammad Rahil Afzal, Misbah Naz, Waqas Ashraf, Daolin Du

**Affiliations:** 1 Institute of Environment and Ecology, School of Environment and Safety Engineering, Jiangsu University, 301 Xuefu Road, Zhenjiang 212013, China; misbahnaz.ray@yahoo.com; 2 Soil and Water Testing Laboratory for Research, Ayub Agricultural Research Institute Faisalabad, Punjab 38850, Pakistan; waqas_ashraf121@yahoo.com

**Keywords:** soil nitrification, plant invasion, legacy effects, mechanisms, context dependence, management, restoration

## Abstract

Plant invasions can have long-lasting impacts on soil nitrification, which plays a critical role in nutrient cycling and plant growth. This review examines the legacy effects of plant invasion on soil nitrification, focusing on the underlying mechanisms, context dependence, and implications for management. We synthesize literature on the positive, negative and neutral legacy effects of plant invasion on soil nitrification, highlighting the complexity of these effects and the need for further research to fully understand them. Positive legacy effects include increased soil microbial biomass or activity, potentially enhancing nutrient availability for plants. However, negative legacy effects, like reduced nitrifier abundance, can result in decreased soil nitrification rates and nutrient availability. In some cases, changes to nitrification during active invasion appear transitory after the removal of invasive plants, indicating neutral short-term legacies. We discuss the context dependence of legacy effects considering factors, including location, specific invasive plant species, and other environmental conditions. Furthermore, we discuss the implications of these legacy effects for management and restoration strategies, such as the removal or control of invasive plants, and potential approaches for restoring ecosystems with legacy effects on soil nitrification. Finally, we highlight future research directions, including further investigation into the mechanisms and context dependence of legacy effects, and the role of plant–microbe interactions. Overall, this review provides insights into the legacy effects of plant invasion on soil nitrification and their implications for ecosystems.

## 1. Introduction

Alien species invading ecosystems are a major driver of global environmental change and the second greatest threat to biodiversity, following habitat loss [1]. These invasive species have significant effects on ecosystem structure and function, including a reduction in diversity of native plant species, alterations in soil nutrient pools and fluxes, and changes in ecosystem productivity [2,3,4]. The ecological impacts of invasive species are extensive and have been focused based on their effects on aboveground terrestrial plant communities’ diversity and dynamics as well as below-ground ecosystem structure and function, specifically the dynamics of the soil system [5,6,7,8]. These studies are particularly relevant because plants and soil have a close relationship, and any alterations in soil properties caused by invasive species can lead to profound changes in vegetation composition and structure [9].

Legacy effects refer to the long-lasting impacts of a disturbance or event on the abiotic and biotic properties of an ecosystem [10]. In the context of soil, legacy effects can result from climate events, land use, and plant growth. These effects may persist for years or even decades and influence soil nutrient cycling, microbial communities, and plant productivity [11]. Plant invasion is a form of disturbance that can also have legacy effects on soil properties. For instance, many invasive plant species release allelopathic chemicals/secondary compounds that can persist in the soil even after the removal of the invasive species and alter the soil nutrient availability, microbial communities, and soil organic matter content [12,13,14,15]. These compounds play a crucial role in promoting the further spread and survival of invasive species [16]. The net effect of these changes on soil nitrification, however, may depend on several factors, such as the invasive plant species, soil type, and the length of time since the invasion [17,18].

Invasive plant species may affect soil nitrogen cycling by altering the nitrogen inputs and outputs, soil pH, and microbial community structure. For example, invasive species can increase soil nitrogen availability and alter soil nitrogen forms by increasing nitrate leaching and decreasing ammonium adsorption [14,17,18]. Invasive plants may also have a significant impact on soil nitrification through their legacy effects. One study found that invasive plants affected soil nitrification rates by altering the soil microbial community and the nitrogen forms available in the soil [17]. Another study investigated the effects of invasive plant species on soil nitrogen cycling and found that invasive species reduced nitrogen mineralization rates and increased nitrate leaching compared to native plants [19]. The study suggested that the reduced nitrogen mineralization rates could be attributed to changes in the soil microbial community and that the increased nitrate leaching may be caused by changes in the plant community.

Overall, the concept of legacy effects is important in understanding the long-term impacts of disturbances on soil properties and ecosystem function. More research is needed to elucidate the specific mechanisms underlying legacy effects and their potential influence on soil nitrification following plant invasion.

## 2. Legacy Effects of Plant Invasion on Soil Nitrification

The legacy effects of plant invasion on soil nitrification allude to the long-lasting influence of invasive plants on the nitrification process in the soil. Nitrification is a microbial-mediated process that drives the conversion of ammonium (NH_4_^+^) into nitrate (NO_3_^−^), which is an essential plant nutrient [20,21,22]. Research studies have shown that the persistence of plant invasion can impact the composition, abundance and the activity of nitrifying microbial communities in the soil, altering nitrogen cycle and the associated ecosystem dynamics [23] (Table 1). These changes can directly influence the soil nitrification rates, which can either increase or decrease depending upon the plant species and their interaction with soil microbiota. Knops et al. [24] explained how plant species traits and differences in general impact nitrogen cycling dynamics in ecosystems based on two main theories, including input-output theory and plant nitrogen use theory. It was concluded that species impacts on nitrogen cycling are not caused by their nitrogen use efficiency but are more strongly related to their impacts on nitrogen inputs and outputs [24]. However, it may not be true in the case of all invasive species, for example, the invasive tree *Pinus strobus* was found to accumulate higher nitrogen and carbon with a longer nitrogen residence time, retaining nitrogen in its tissues for longer periods compared to native grasses and oak trees. This allowed *P. strobus* to accumulate more nitrogen from the soil over time without an equal return through decomposition [25].

Invasive plants frequently produce significant amounts of leaf litter and release root exudates, which can alter the pH and nutrient availability and reshape the microbial community structure in the soil system over time [23,26]. These plant inputs consist of both aboveground and belowground mechanisms. The aboveground litter, including fallen leaves, upon decomposition, can serve as a source of organic matter (OM) and nutrients for the soil. Moreover, decomposing leaf litter and roots release compounds that acidify or alkalize soils based on their chemical composition [27]. The decomposition rate is mainly attributed to the chemical composition of invasive plant litter.

**Table 1:** Changes in the nitrification associated with plant invasions.

Plant Species	Study Location	Impact on Soil Nitrification	Mechanisms	References
*Avena barbata*, *Bromus hordeaceus*, and *Lupinus bicolor*	California grassland, USA	Increasing	Increasing the abundance and changing the composition of ammonia-oxidizing bacteria in soil.	[28]
*Mikania micrantha*	Guangdong, China	Increasing	Increased abundance of nitrification genes, including AOA, AOB, and AOC genes.	[29]
*Bidens alba*	Guangzhou, China	Increasing	Increased abundance of ammonia-oxidizing bacteria (AOB) and ammonia-oxidizing archaea (AOA) in the soil.	[30]
*Ambrosia artemisiifolia* and *Bidens pilosa*	Beijing, China	Decreasing	Altered soil microbial community structure and function with low relative abundances of functional genes related to nitrification.	[31]
moso bamboo (*Phyllostachys edulis*)	Jiangxi, China	Decreasing	Decreased net nitrification rates with N addition and increases in denitrification, leading to increased N_2_O emissions.	[32]
*Aegilops triuncialis* and *Elymus caput-medusae*	California, USA	Increasing	Alteration of microbial community composition and diversity.	[33]
*Microstegium vimineum*	Indiana/USA	Increasing	Altered soil nitrogen cycling associated with higher soil pH by promoting the conversion of ammonia to nitrate through the process of nitrification.	[34]
*Mikania micrantha*	Guangzhou, China	Increasing	Changes in soil microbial communities and nutrient availability.	[35]
*Wedelia trilobata*, *Ipomoea cairica* and *Mikania micrantha*	Southern China	Decreasing for *Wedelia trilobata* and *Ipomoea cairica* Both increasing and and decreasing for *Mikania micrantha*	The mechanisms of invasion of the studied invasive species remained unclear; however, it was suggested that the chemical composition of leaf leachates may be a cause of the changes observed in soil nitrogen transformation and the abundance of ammonia oxidizers.	[36]
*Microstegium vimineum*	Indiana, USA	No difference in potential nitrification rates after removal	Recovery of nitrification function.	[37]
*Bromus tectorum*	Colorado, USA	No difference in nitrifier abundance after removal	Resilience of nitrifier communities.	[38]
*Acacia saligna*	South Africa	No difference in potential nitrification rates after removal	Functional resilience of nitrifier communities.	[39]

Several studies report that litter from invasive plants decomposes faster than that of native plants, resulting in an accelerated nutrient cycling rate and release of organic acids, reducing soil pH in invaded systems [27,40,41,42]. For instance, the invasive herb *Alliaria petiolate* produces litter high in calcium that contributes to increased nitrate leaching and soil acidification in North American hardwood forests [43]. Acidic soils favor fungal-driven nitrification pathways [21]. Such variations in soil pH resulting from plant invasions might shift the balance of fungal and bacterial dominance and provide favorable niches for certain nitrifiers over others, causing a long-term alteration in community structure and function [34]. However, some studies also indicate that the invasive plant litter decomposition may be slower than native plant litter decomposition. The invasive species *Phragmites australis*, for instance, had a slower rate of aboveground litter decomposition compared to the native plant *Spartina patens* [44]. In addition, *Aegilops triuncialis*, an invasive herbaceous plant, has been shown to reduce nitrogen cycling in the invaded area by slowing litter decomposition [43]. The reduced rate of litter decomposition could be attributed to higher lignin and polyphenol content in the litter [45]. However, the influence of invasive plants on nitrogen cycling depends not just on the dynamics of their own litter, but also on complex synergistic interactions between invasive and native litter that can accelerate overall decomposition and nitrogen release in the invaded ecosystem [46,47,48,49].

The belowground inputs, for example root litter and exudates, can significantly impact the soil microbiota by acting as a source of carbon for their growth and influencing their activity involved in the process of nitrification [23]. Several studies have reported that the allelochemicals present in the root exudates of invasive plants showed significant impacts on the structure and function of soil microbial communities [15,50,51,52,53,54,55]. For example, catechin from invasive *Centaurea maculosa* and *Centaurea* stobe in North America significantly impacted the number of cultivable bacteria and reduced soil nitrification rates, respectively [56,57]. Soil N transformation processes and the structure and function of soil microorganisms have been shown to be significantly altered by the allelopathic effects of invasive plants, but the impact of these changes on the number, composition, structure, and function of ammonia-oxidizing microorganisms has received less attention and needs to be further investigated [58,59,60]. However, the root exudates of other plant species, including tropical grasses like *Brachiaria humidicola* and some cereals like sorghum, contain nitrification inhibitors termed ‘biological nitrification inhibitors’ (BNIs) that suppress the activity of nitrifying bacteria like *Nitrosomonas* spp. in the soil [61,62,63]. The inhibitors include chemicals like hydrophilic brachialactone and phenylpropanoids, and hydrophobic sorgoleone that can block enzymes critical for nitrification, like ammonia monooxygenase and hydroxylamine oxidoreductase in Nitrosomonas [61,62]. By suppressing nitrifier activity, BNI plants can substantially reduce nitrification rates and ammonium oxidation in soils, leading to smaller nitrifier populations over time [62]. Studies showed 90% declines in soil ammonium oxidation rates in fields planted with Brachiaria humidicola compared to other species. The production and release of BNIs by plant roots is triggered by the presence of ammonium in the soil, and the strength of nitrification inhibition can differ based on plant genetics [63,64]. It has been reported that the transport of hydrophilic BNIs may take place via anion channels, ABC transporters, or MATE proteins powered by plasma membrane H^+^-ATPase activity [65,66,67]. On the other hand, the release of hydrophobic BNIs is independent of the proton motive force or membrane potential generated by H^+^-ATPase [65] and may involve vesicle trafficking and exocytosis [68]. This localized and targeted inhibition of nitrifiers likely helps retain nitrogen and reduces losses. Not all potential BNIs released by plants are equally effective in soils. Compounds like brachialactone seem more persistent and suppressive compared to others [69]. It has been suggested that plants have evolved BNI functions as an adaptation to conserve nitrogen in low nitrogen environments [63]. Despite biodegradability, these allelochemicals and BNIs present a sustained selective pressure on nitrifiers during invasion. After invaders are eliminated, the reduction in nitrifier abundance and alteration in community structure may persist for years, providing a chemical legacy effect on nitrification [70].

The changes in the soil microbial communities and nutrient processes induced by the invasive plants can produce lasting effects on nitrification, even after their removal from the soil, and can affect the growth and functions of the succeeding plant species inhabiting the invaded area for an extended period [28]. The soil legacy effects may act as a feedback loop whereby the alterations in soil microbial communities resulting from the non-native plants can impact the growth and establishment of future plant species [71]. 

**Figure 1:**
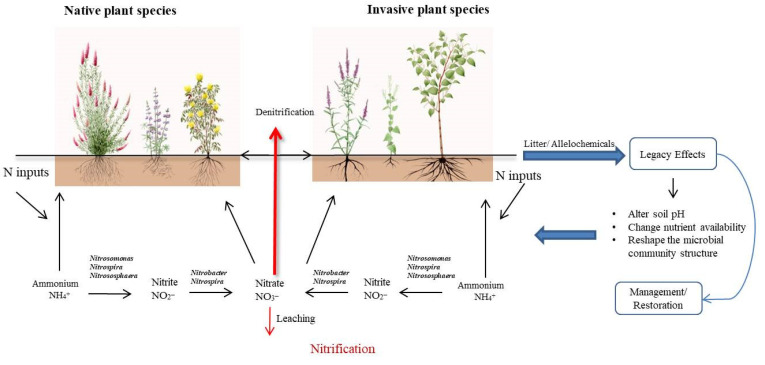
The figure shows the mechanism of legacy effects of invasive plants on the soil nitrification.

It is noteworthy that the legacies of plant invasion on soil nitrification are contingent upon the particular invasive plant species, the attributes of the invaded ecosystem, and the interplay between the invasive flora and the indigenous soil microbial communities [72,73]. Furthermore, there is still a lack of complete comprehension regarding the impacts of aboveground and belowground input components on the soil nitrification and the microbial communities involved [23]. More investigation is required to elucidate the mechanisms underlying these legacy effects and their implications for the ecosystem functioning. Understanding the legacy effects is of utmost importance in the effective management and restoration of invaded ecosystems, as well as in the preservation of the ecological equilibrium between plants and soil microbial communities (Figure 1). 

### 2.1. Positive Legacy Effects of Plant Invasion on Soil Nitrification

Invasive species can have positive effects on soil nitrification that leads to higher microbial biomass and activity in the soil. These effects have a significant contribution towards soil nutrient availability and ultimately the plant growth. For example, nitrogen-fixing invasive species can enhance the availability of nutrients and have a beneficial impact on the growth of recolonizing plant species, particularly those that grow rapidly and can take advantage of the increased nutrient availability [74].

Geddes et al. [75] found that the older invaded parts of the chronosequence had bigger soil nutrient pools and diversity of denitrifiers in comparison to uninvaded areas, implying that the invasive plants may have positive legacy effects on soil nitrification. According to the findings of the study, soil nutrient pools (organic matter, nitrate, and ammonium), as well as the diversity of denitrifiers, were greater in the older Typha × glauca-invaded areas of the chronosequence. This lends credence to the contention that age since invasion and persistent soil “legacies” are important factors to take into consideration when researching the effects of invaders on ecosystems. Invasive plants can modify soil microbial populations, with consequences for ecological processes that may be an invisible legacy of non-native plant invasions. A study reported that invasive annual grasses in a grassland system in California could potentially gain advantages from changes in the nitrogen cycle within the soil [29]. These grasses have the ability to enhance the overall rates of nitrification and modify the types of bacteria responsible for ammonia oxidation in the soil. Consequently, this could result in a shift in the nitrogen balance of the ecosystem following the invasion. Conversely, the native grasses did not exhibit a similar rise in gross nitrification rates observed in the invasive grasses. This process of self-promotion facilitated by plant–microbe interactions serves as a mechanism through which invasive plants can effectively invade and establish themselves in new environments. From a functional perspective, increased nitrogen absorption is expected to have a beneficial impact on the fitness of invasive grasses, enabling them to sustain their dominant role within the community [36]. Stark and Norton [76] discussed the positive legacy effects of cheatgrass on soil nitrification. The study showed that *Bromus tectorum* L. (cheatgrass), compared to *Artemisia tridentata* Nutt. (sagebrush), perennial grass stands of the same age, enhances the availability of nitrogen in the soil. Cheatgrass dominance leads to higher rates of both net and gross nitrogen mineralization, as well as net nitrification. The study demonstrates that cheatgrass soils, whether disturbed or intact, exhibited increased net rates of nitrogen cycling during field and laboratory incubations. Furthermore, cheatgrass soils contained higher concentrations of soil organic carbon and nitrogen compared to sagebrush-grass soils, suggesting that cheatgrass has beneficial long-term effects on soil nitrification. A study also demonstrates that invasive species like *Microstegium vimineum* enhance nitrification rates in the invaded soil, and it proposes that monocultures of invasions are sustained by the presence of high soil nitrate concentrations. These positive plant–soil interactions, facilitated by microbial nutrient transformations and nutrient availability, may be an underrecognized mechanism that supports the persistence of plant invasions [77].

In summary, positive legacy effects of plant invasion on soil nitrification can arise from changes in soil microbial communities, nutrient availability, and nutrient cycling dynamics caused by invasive plants. These effects include the establishment of novel plant–soil feedbacks, increased nutrient release from decomposers, and persistent microbial soil legacies.

### 2.2. Negative Legacy Effects of Plant Invasion on Soil Nitrification

Invasive plants can have negative legacies on the process of soil nitrification by altering the soil nitrifying community and impacting the soil nutrients [78]. These legacy effects can influence growth and successional pathways of the new plant community. Amatangelo et al. [79] found that the impacts of removing litter one year later on plant available nitrogen and microbial community composition can persist for at least another year. The litter produced by highly invaded populations had an indirect negative effect on soil nutrients, proving that invasive systems can be controlled by adjusting soil nutrient levels. The opposite was found to be true with non-invaded communities where the litter appeared to facilitate the annual grasses production, which could hasten the rate at which desired species are displaced by invasive ones [80]. During fast vegetation changes like exotic species invasions or native species restoration, the legacy effect of long-term litter inputs from invasive plants outweighs the short-term effects [23]. The legacy effect of the preceding plant type determines the soil microbial community structure and function, not the current vegetation. These detrimental legacy effects can last for extended periods, even in the face of restoration endeavors. A study by Carey et al. [81] showed that two invasive plant species, *Aegilops triuncialis* (goatgrass) and *Elymus caput-medusae* (Medusahead), decreased soil NO_3_^−^ and potential nitrification as compared to native plant communities in California. This indicates a negative impact on soil nitrification caused by invasive plants, and these changes in soil conditions may contribute to the persistence of invasive plant populations, potentially hindering restoration efforts. A study by Suding et al. [82] revealed that plots invaded by non-native grasses experienced reduced soil nitrogen availability and rates of net nitrification compared to uninvaded plots. These effects were observed for a minimum of two years following the removal of the invasive grasses. Similarly, Hawkes et al. [83] reported that the plots invaded by cheatgrass (*Bromus tectorum*) when introduced into sagebrush ecosystems exhibited lower soil nitrogen availability and rates of net nitrification compared to uninvaded plots, and these impacts persisted for at least three years following the removal of the invasive grasses.

Invasive plants have been reported to have negative legacy effects on soil microbial communities and nitrogen cycling, which may survive even after the treatment of herbicide [28]. The study showed that nitrifiers were found in short supply at both herbicide-treated and highly invaded untreated-sites, suggesting legacy consequences in nitrogen cycling. Given the continued lack of nitrifiers in herbicide-treated areas, nitrogen-fixing species like bush lupine may have an advantage in colonizing these areas, which could prevent the recovery of native vegetation and cast doubt on the success of restoration efforts. In contrast, native plants may have a positive effect on soil nitrification. A study in California found that the abundance of nitrifiers was higher in native-dominated sites compared to non-native-dominated sites [84]. The study suggests that this difference may be due to the higher quality of organic matter inputs from native plants, which may promote the growth of nitrifiers. Additionally, the study found that the abundance of nitrifiers was positively correlated with the abundance of native plant species, indicating that the presence of native plants may enhance soil nitrification. It was proposed that the presence of invasive plants can have adverse consequences on soil nitrification, leading to a decline or even complete loss of vital ecosystem processes like denitrification when these aggressive invaders establish themselves over a long duration [75]. The research discovered that the newly invaded site exhibited the highest denitrification rates, suggesting that when aggressive invaders take root at a location for extended periods, significant ecosystem functions like denitrification may diminish or ultimately vanish.

It is important to note that the specific negative effects of invasive plants on soil nitrification can vary depending on the particular invasive species, ecosystem characteristics, and management practices. These effects can have significant implications for ecosystem functioning, nutrient cycling, and plant community dynamics.

### 2.3. Neutral Legacy Effects of Plant Invasion on Soil Nitrification

Invasive plants can have neural effects on soil nitrification rates, nitrifier abundance, and community composition over the long-term following invasion and removal, indicating a lack of persistent positive or negative legacy impacts on the nitrification process in the soils of the corresponding ecosystems. Studies on the neutral legacy effects of plant invasions on soil nitrification have reported mixed results, with some demonstrating transient effects but others reporting persistent alterations even after invader removal. Overall, research on the processes and context-dependence of neutral nitrification legacies is limited in comparison to negative or positive impacts [85].

A study in Hawaii investigated the legacy effects of the nitrogen-fixing invasive tree *Morella faya* 5–7 years after its removal from the invaded site [74]. It was found that the potential net nitrification rates, ammonia-oxidizing archaea abundance, and bacterial and archaeal ammonia oxidizer community composition returned to levels statistically similar to uninvaded sites after the removal of *M. faya*. The study concluded that the positive legacy effects of this invasive N-fixer on nitrification were reversible once it was removed from the ecosystem, indicating that the removal of *M. faya* allowed recovery of the nitrifier community and nitrification function to a non-invaded state. Several recent studies have documented a lack of legacy effects on nitrifier abundance or nitrification rates in historically invaded soils compared to uninvaded soils [85]. However, the emerging picture is mixed. A global meta-analysis found negligible legacy effects in most cases, but substantial variation across invasive plant species [85]. They propose neutral legacies may arise when invaders minimally alter resource availability for nitrifiers. In contrast, another study showed plant litter inputs could impose lasting legacy effects on microbial communities and nitrogen cycling for up to 3 years post-removal in grasslands [71]. This suggests species that modify soil habitat can impart persistent impacts even after elimination. While transitory effects have been documented in some cases, the mechanisms driving neutral legacy emergence and persistence remain unclear. Studies have postulated various factors like invasion duration, removal efficacy, resource competition, and environmental context may interact to determine legacy strength [71]. Elucidating these mechanisms is an important frontier for invasion legacy research.

In these cases, changes to the nitrifier community and nitrification processes induced during active invasion appeared transitory once the invasive species was removed from the ecosystem. Within a few years, aspects of the nitrification function like nitrate production rates and nitrifier community structure appeared resilient and recovered to conditions similar to never-invaded soils. The timescale of these studies may not have been long enough to fully evaluate legacy effects, which could take decades to disappear in some ecosystems. However, the evidence does suggest neutral or negligible legacy impacts on nitrification for some invasive plant species over this shorter time span. Further investigation across diverse ecosystems is needed to discern when neutral legacies will emerge and their implications for restoration.

## 3. Context Dependence of Legacy Effects on Soil Nitrification

The legacy effects of invading plants on soil nitrification might vary greatly depending on the location of the invasion, the particular species of invasive plant that was involved, and any other relevant environmental conditions [86]. The presence of invasive plant species can cause major changes to the characteristics of the soil and the microbial communities inside, which in turn can cause shifts in the processes that regulate the cycling of nutrients and the functioning of ecosystems.

It is possible for the legacy effects on soil nitrification to be affected by the location where the invasive plant species first establish themselves. The qualities of the soil, the meteorological circumstances, as well as the existing plant and microbial communities, might differ from one area to another, which can lead to changes in the processes of soil nitrification. For instance, variations in soil pH, the amount of organic matter present, and the availability of nutrients can all have an effect on the activity and composition of nitrifying microorganisms, which in turn can have an effect on the rates of nitrification in the soil [87,88]. In addition, environmental conditions such as temperature and precipitation can have an effect on plant growth, litter breakdown, and microbial activity, all of which can indirectly have an effect on soil nitrification [71,73].

How the legacies of plant invasion impact on soil nitrification are also determined by the specific invasive plants species involved. Various invasive plant species exhibit a wide variety of characteristics and interactions with the microbial communities in the soil, all of which have the potential to affect nitrification in the soil. Invasive plants have the potential to either release allelochemicals or affect the environment of the rhizosphere, both of which have the potential of changing the composition and activity of the soil microbiota that are engaged in the nitrification process. Depending on the particular plant–microbe interactions, these shifts can either boost or lower the soil’s nitrification rates [85,88,89]. Furthermore, invasive plants may have distinct strategies for nutrient uptake, variable rates of litter decomposition, or various root exudation patterns, all of which further influence the soil’s nutrient cycle and nitrification [16,90].

The residual impacts of invasive plants on soil nitrification can also be influenced by other environmental factors, such as the availability of nutrients and the disturbances in the ecosystem. The growth and competitive advantage of invasive plants can be affected by the availability of nutrients, particularly nitrogen deposition, which has the potential to change the effect that these plants have on nitrification in the soil [18,91]. The environment of the soil can be altered by disturbances such as fire or the history of land use, which can have an effect not only on the establishment of invasive plant species, but also on the functioning of nitrifying microorganisms and, as a result, on the processes of nitrification in the soil [10,92].

Having provided the above information, there is still a need to explore the combined influence of location, invasive plant species, and other environmental factors on soil nitrification. However, considering the general understanding of the topic and the factors involved, it is reasonable to assume that these factors interact and collectively shape the legacy effects of invasive plants on soil nitrification. Understanding these effects can provide insights into the long-term implications of plant invasions on soil ecosystems and help improve predictions and management strategies for invasive species.

## 4. Implications for Management and Restoration

Invasive plant species have the potential to have a substantial impact on soil nitrification, impacting nitrogen cycling, ecosystem processes, and native plant communities. Managing invasive plant species based on their effects on soil nitrification necessitates a complete approach that takes into account the invasive species’ particular traits, the local ecology, and available resources.

The elimination and management of invasive plant species is one method for dealing with the problem. This may require repeated burning, grazing, pruning, mowing, or uprooting the plants, either by hand or with a machine, and could be incorporated when reviewing soil nitrogen management options [93]. When invasive plants are eradicated, native plant species are given a better chance to flourish because they face less competition for limited nutrients like nitrogen. Changes in plant community composition and nutrient dynamics may result in unintended consequences for soil nitrification [93,94]. In addition, to control invasive species and restore degraded ecosystems, manipulating soil nutrients, particularly nitrogen, can be an effective method. Reducing the availability of nitrogen through techniques such as nitrogen immobilization may reduce the invasiveness of nutrient-rich ecosystems [93]. Methods like carbon addition and establishing native plants that conserve nitrogen can lower nitrogen, but can also be costly to implement and maintain, or have only temporary effects [95,96,97,98,99,100,101]. According to studies, reducing nitrogen levels can reduce the biomass and development of invasive species such as *Phragmites australis* while encouraging the growth of native species such as *Melaleuca ericifolia* [102]. Furthermore, it has been reported that native perennial grasses were better able to compete with invasive annual grasses at low soil nitrogen levels. This supports the concept that lowering soil nitrogen can help shift competitive advantage to native species over invasives [93,103]. This also proposes a conceptual model predicting community dynamics based on soil nitrogen levels, where native species dominate at low nitrogen but invasives dominate at higher nitrogen. This model could be referenced when discussing management implications of altering soil nitrogen. In addition, a global meta-analysis suggests that managing nitrogen must be paired with priority effect and propagule management tools, including herbicide, mowing, and grazing for effective restoration strategies [103].

Topsoil removal rapidly and dramatically lowers nitrogen availability by extracting the surface soil layers where nitrogen accumulates [104]. However, it also involves intense disturbance that may inhibit re-establishment of native species [105]. It may only be suitable in systems adapted to major sod disturbance, like certain grasslands or heathlands undergoing restoration. The potential for managers to control soil nitrogen availability depends heavily on the current nitrogen inputs to the ecosystem from atmospheric deposition, agricultural runoff, or other sources [106,107]. Management efforts are more likely to succeed in ecosystems that are not subject to high ongoing external nitrogen loading. Where feasible, reducing excessive nitrogen inputs will expand opportunities for management strategies aimed at lowering soil nitrogen. When implementing any nitrogen management strategy, it is critical to consider broader ecological impacts besides nitrogen availability, such as effects on non-target native species. Adaptive monitoring and assessment is essential to gauge whether the benefits of nitrogen control methods outweigh any negative consequences.

Ecosystems that have been subjected to legacy effects on soil nitrification may necessitate the implementation of specific restoration strategies to address the altered nitrogen dynamics. Introducing beneficial microorganisms to the soil can aid in the restoration of nitrification processes. Certain microbial communities play a crucial role in nitrification, and their absence or alteration due to disturbances can disrupt nitrogen cycling. By inoculating the soil with nitrifying microorganisms, the restoration of nitrification can be promoted, helping to reestablish the natural nutrient cycling processes [102]. Applying nutrient amendments, such as organic matter or specific fertilizers, can help restore nitrogen availability and promote nitrification. Organic matter additions can improve soil fertility and provide a source of nitrogen for plant uptake and microbial activity. Controlled application of fertilizers, considering the specific nutrient requirements and limitations of the ecosystem, can also aid in restoring soil nitrification processes [102].

It is important to note that the specific management and restoration strategies may vary depending on the characteristics of the invasive species, the ecosystem, and local conditions. Monitoring and adaptive management approaches are essential to assess the effectiveness of these strategies and make adjustments as necessary.

## 5. Future Research Directions

The legacy effects of plant invasion on soil nitrification are a complex phenomenon that requires further research to fully understand the underlying mechanisms and context dependence. Non-native plants have the potential to significantly modify soil microbial communities [23]. These alterations can influence soil nutrient processes, including nitrification. Understanding the specific mechanisms by which invasive plants affect soil microbial communities and their subsequent impact on nitrification is crucial.

There is still a lack of consensus regarding the relative effects of aboveground and belowground plant inputs on nutrient cycling and microbial communities [23,108,109]. Determining whether aboveground inputs (e.g., leaf litter) or belowground inputs (e.g., root litter and exudates) have a primary influence on soil nitrification is essential for comprehending the overall dynamics of legacy effects. Moreover, considering the spatial variability of plant invasion impacts on soil nitrification is crucial for capturing the full context dependence.

## 6. Conclusions

In conclusion, the impact of plant invasions on soil nitrification is a complex and multifaceted phenomenon. The invasion of alien plant species can have both short-term and long-term effects on soil microbial community composition, nutrient cycling processes, and soil carbon and nitrogen content. Understanding the legacy of plant invasions on soil nitrification is crucial for effective management and restoration strategies, as it provides insights into the mechanisms driving ecosystem changes and the potential implications for ecosystem functioning and services. Further research is needed to explore the long-term consequences of plant invasions on soil nitrification and develop comprehensive management approaches that consider the complex interactions between invasive plants, soil microbial communities, and nutrient dynamics.

## Data Availability

There is no data availability statement.

## References

[1] Simberloff D., Martin J.-L., Genovesi P., Maris V., Wardle D.A., Aronson J., Courchamp F., Galil B., García-Berthou E., Pascal M. (2013). Impacts of biological invasions: What’s what and the way forward. Trends Ecol. Evol..

[2] Liao C., Peng R., Luo Y., Zhou X., Wu X., Fang C., Chen J., Li B. (2008). Altered ecosystem carbon and nitrogen cycles by plant invasion: A meta-analysis. New Phytol..

[3] Ahmad R., Khuroo A.A., Hamid M., Rashid I. (2019). Plant invasion alters the physico-chemical dynamics of soil system: Insights from invasive Leucanthemum vulgare in the Indian Himalaya. Environ. Monit. Assess..

[4] Tekiela D.R., Barney J.N. (2017). Invasion shadows: The accumulation and loss of ecological impacts from an invasive plant. Invasive Plant Sci. Manag..

[5] Hejda M., Pyšek P., Jarošík V. (2009). Impact of invasive plants on the species richness, diversity and composition of invaded communities. J. Ecol..

[6] Meffin R., Miller A.L., Hulme P.E., Duncan R.P. (2010). Biodiversity research: Experimental introduction of the alien plant Hieracium lepidulum reveals no significant impact on montane plant communities in New Zealand. Divers. Distrib..

[7] Čuda J., Vitkova M., Albrechtova M., Guo W.-Y., Barney J.N., Pyšek P. (2017). Invasive herb Impatiens glandulifera has minimal impact on multiple components of temperate forest ecosystem function. Biol. Invasions.

[8] Baranová B., Fazekašová D., Manko P. (2017). Variations of selected soil properties in the grass fields invaded and uninvaded by invasive goldenrod (*Solidago canadensis* L.). Ekológia.

[9] Timsina B., Shrestha B.B., Rokaya M.B., Münzbergová Z. (2011). Impact of *Parthenium hysterophorus* L. invasion on plant species composition and soil properties of grassland communities in Nepal. Flora-Morphol. Distrib. Funct. Ecol. Plants.

[10] Cuddington K. (2011). Legacy effects: The persistent impact of ecological interactions. Biol. Theory.

[11] Nguyen L.T., Osanai Y., Anderson I.C., Bange M.P., Tissue D.T., Singh B.K. (2018). Flooding and prolonged drought have differential legacy impacts on soil nitrogen cycling, microbial communities and plant productivity. Plant Soil.

[12] Jordan N.R., Larson D.L., Huerd S.C. (2008). Soil modification by invasive plants: Effects on native and invasive species of mixed-grass prairies. Biol. Invasions.

[13] Wardle D.A., Karban R., Callaway R.M. (2011). The ecosystem and evolutionary contexts of allelopathy. Trends Ecol. Evol..

[14] Ehrenfeld J.G., Kourtev P., Huang W. (2001). Changes in soil functions following invasions of exotic understory plants in deciduous forests. Ecol. Appl..

[15] Lorenzo P., Pereira C.S., Rodríguez-Echeverría S. (2013). Differential impact on soil microbes of allelopathic compounds released by the invasive Acacia dealbata Link. Soil Biol. Biochem..

[16] Zhang Z., Bhowmik P.C., Suseela V. (2021). Effect of soil carbon amendments in reversing the legacy effect of plant invasion. J. Appl. Ecol..

[17] Cavagnaro T.R. (2016). Soil moisture legacy effects: Impacts on soil nutrients, plants and mycorrhizal responsiveness. Soil Biol. Biochem..

[18] Ehrenfeld J.G. (2003). Effects of exotic plant invasions on soil nutrient cycling processes. Ecosystems.

[19] Mok H.F., Arndt S.K., Nitschke C.R. (2012). Modelling the potential impact of climate variability and change on species regeneration potential in the temperate forests of S outh-E astern A ustralia. Glob. Change Biol..

[20] Kozlowski J.A., Stieglmeier M., Schleper C., Klotz M.G., Stein L.Y. (2016). Pathways and key intermediates required for obligate aerobic ammonia-dependent chemolithotrophy in bacteria and Thaumarchaeota. ISME J..

[21] Norton J., Ouyang Y. (2019). Controls and adaptive management of nitrification in agricultural soils. Front. Microbiol..

[22] Schleper C., Nicol G.W. (2010). Ammonia-oxidising archaea–physiology, ecology and evolution. Adv. Microb. Physiol..

[23] Elgersma K.J., Ehrenfeld J.G., Yu S., Vor T. (2011). Legacy effects overwhelm the short-term effects of exotic plant invasion and restoration on soil microbial community structure, enzyme activities, and nitrogen cycling. Oecologia.

[24] Knops J., Bradley K., Wedin D. (2002). Mechanisms of plant species impacts on ecosystem nitrogen cycling. Ecol. Lett..

[25] Laungani R., Knops J.M. (2009). Species-driven changes in nitrogen cycling can provide a mechanism for plant invasions. Proc. Natl. Acad. Sci. USA.

[26] Li J., Leng Z., Wu Y., Du Y., Dai Z., Biswas A., Zheng X., Li G., Mahmoud E.K., Jia H. (2022). Interactions between invasive plants and heavy metal stresses: A review. J. Plant Ecol..

[27] Rothstein D.E., Vitousek P.M., Simmons B.L. (2004). An exotic tree alters decomposition and nutrient cycling in a Hawaiian montane forest. Ecosystems.

[28] Parsons L.S., Sayre J., Ender C., Rodrigues J.L., Barberán A. (2020). Soil microbial communities in restored and unrestored coastal dune ecosystems in California. Restor. Ecol..

[29] Hawkes C.V., Wren I.F., Herman D.J., Firestone M.K. (2005). Plant invasion alters nitrogen cycling by modifying the soil nitrifying community. Ecol. Lett..

[30] Zhao P., Liu B., Zhao H., Lei Z., Zhou T. (2023). Significant changes in soil microbial community structure and metabolic function after Mikania micrantha invasion. Sci. Rep..

[31] Wei H.-J., Chen B.-M. (2023). Competition shifts the advantage of the invasive plant Bidens alba to a disadvantage under soil ammonia nitrogen. Biol. Invasions.

[32] Wang L., Li Q., Li C., Wu C., Chen F., Chen X., Zhang F. (2023). Nitrate Nitrogen and pH Correlate with Changes in Rhizosphere Microbial Community Assemblages during Invasion of Ambrosia artemisiifolia and Bidens pilosa. Microbiol. Spectr..

[33] Li Z., Zhang L., Deng B., Liu Y., Kong F., Huang G., Zou Q., Liu Q., Guo X., Fu Y. (2017). Effects of moso bamboo (Phyllostachys edulis) invasions on soil nitrogen cycles depend on invasion stage and warming. Environ. Sci. Pollut. Res..

[34] Carey C.J., Beman J.M., Eviner V.T., Malmstrom C.M., Hart S.C. (2015). Soil microbial community structure is unaltered by plant invasion, vegetation clipping, and nitrogen fertilization in experimental semi-arid grasslands. Front. Microbiol..

[35] Shannon-Firestone S., Reynolds H.L., Phillips R.P., Flory S.L., Yannarell A. (2015). The role of ammonium oxidizing communities in mediating effects of an invasive plant on soil nitrification. Soil Biol. Biochem..

[36] Yu H., Le Roux J.J., Jiang Z., Sun F., Peng C., Li W. (2021). Soil nitrogen dynamics and competition during plant invasion: Insights from Mikania micrantha invasions in China. New Phytol..

[37] Brewer J.S. (2011). Per capita community-level effects of an invasive grass, Microstegium vimineum, on vegetation in mesic forests in northern Mississippi (USA). Biol. Invasions.

[38] Perkins L.B., Nowak R.S. (2012). Soil conditioning and plant–soil feedbacks affect competitive relationships between native and invasive grasses. Plant Ecol..

[39] Yelenik S.G., Stock W.D., Richardson D.M. (2007). Functional group identity does not predict invader impacts: Differential effects of nitrogen-fixing exotic plants on ecosystem function. Biol. Invasions.

[40] Allison S.D., Vitousek P.M. (2004). Rapid nutrient cycling in leaf litter from invasive plants in Hawai’i. Oecologia.

[41] CHEN B.-M., WEI H.-J., CHEN W.-B., ZHU Z.-C., YUAN Y.-R., ZHANG Y.-L., LAN Z.-G. (2018). Effects of plant invasion on soil nitrogen transformation processes and its associated microbes. Chin. J. Plant Ecol..

[42] Meisner A., De Boer W., Cornelissen J.H., van der Putten W.H. (2012). Reciprocal effects of litter from exotic and congeneric native plant species via soil nutrients. PLoS ONE.

[43] Rodgers V.L., Wolfe B.E., Werden L.K., Finzi A.C. (2008). The invasive species Alliaria petiolata (garlic mustard) increases soil nutrient availability in northern hardwood-conifer forests. Oecologia.

[44] Windham L., Ehrenfeld J.G. (2003). Net impact of a plant invasion on nitrogen-cycling processes within a brackish tidal marsh. Ecol. Appl..

[45] Godoy O., Castro-Díez P., Van Logtestijn R.S., Cornelissen J.H., Valladares F. (2010). Leaf litter traits of invasive species slow down decomposition compared to Spanish natives: A broad phylogenetic comparison. Oecologia.

[46] Chapman S.K., Newman G.S., Hart S.C., Schweitzer J.A., Koch G.W. (2013). Leaf litter mixtures alter microbial community development: Mechanisms for non-additive effects in litter decomposition. PLoS ONE.

[47] Chen B.-M., Peng S.-L., D’Antonio C.M., Li D.-J., Ren W.-T. (2013). Non-additive effects on decomposition from mixing litter of the invasive Mikania micrantha HBK with native plants. PLoS ONE.

[48] Finerty G.E., de Bello F., Bílá K., Berg M.P., Dias A.T., Pezzatti G.B., Moretti M. (2016). Exotic or not, leaf trait dissimilarity modulates the effect of dominant species on mixed litter decomposition. J. Ecol..

[49] Scherer-Lorenzen M. (2008). Functional diversity affects decomposition processes in experimental grasslands. Funct. Ecol..

[50] Cipollini D., Rigsby C.M., Barto E.K. (2012). Microbes as targets and mediators of allelopathy in plants. J. Chem. Ecol..

[51] Czaban W., Rasmussen J., Laursen B.B., Vidkjær N.H., Sapkota R., Nicolaisen M., Fomsgaard I.S. (2018). Multiple effects of secondary metabolites on amino acid cycling in white clover rhizosphere. Soil Biol. Biochem..

[52] Ni G., Song L., Zhang J., Peng S. (2006). Effects of root extracts of Mikania micrantha HBK on soil microbial community. Allelopath. J..

[53] Van der Putten W.H. (2010). Impacts of soil microbial communities on exotic plant invasions. Trends Ecol. Evol..

[54] Watkins A.J., Nicol G.W., Shaw L.J. (2009). Use of an artificial root to examine the influence of 8-hydroxyquinoline on soil microbial activity and bacterial community structure. Soil Biol. Biochem..

[55] Zhu X., Li Y., Feng Y., Ma K. (2017). Response of soil bacterial communities to secondary compounds released from Eupatorium adenophorum. Biol. Invasions.

[56] Bais H.P., Vepachedu R., Gilroy S., Callaway R.M., Vivanco J.M. (2003). Allelopathy and exotic plant invasion: From molecules and genes to species interactions. Science.

[57] Thorpe A.S., Callaway R.M. (2011). Biogeographic differences in the effects of Centaurea stoebe on the soil nitrogen cycle: Novel weapons and soil microbes. Biol. Invasions.

[58] Bradley R., Titus B., Preston C. (2000). Changes to mineral N cycling and microbial communities in black spruce humus after additions of (NH4) 2SO4 and condensed tannins extracted from Kalmia angustifolia and balsam fir. Soil Biol. Biochem..

[59] Joanisse G., Bradley R., Preston C., Munson A. (2007). Soil enzyme inhibition by condensed litter tannins may drive ecosystem structure and processes: The case of Kalmia angustifolia. New Phytol..

[60] Uddin M.N., Robinson R.W., Buultjens A., Al Harun M.A.Y., Shampa S.H. (2017). Role of allelopathy of Phragmites australis in its invasion processes. J. Exp. Mar. Biol. Ecol..

[61] Subbarao G., Ito O., Sahrawat K., Berry W., Nakahara K., Ishikawa T., Watanabe T., Suenaga K., Rondon M., Rao I.M. (2006). Scope and strategies for regulation of nitrification in agricultural systems—Challenges and opportunities. Crit. Rev. Plant Sci..

[62] Subbarao G., Nakahara K., Hurtado M.d.P., Ono H., Moreta D., Salcedo A.F., Yoshihashi A., Ishikawa T., Ishitani M., Ohnishi-Kameyama M. (2009). Evidence for biological nitrification inhibition in Brachiaria pastures. Proc. Natl. Acad. Sci. USA.

[63] Subbarao G., Rondon M., Ito O., Ishikawa T., Rao I.M., Nakahara K., Lascano C., Berry W. (2007). Biological nitrification inhibition (BNI)—Is it a widespread phenomenon?. Plant Soil.

[64] Subbarao G., Sahrawat K.L., Nakahara K., Ishikawa T., Kishii M., Rao I., Hash C., George T., Rao P.S., Nardi P. (2012). Biological nitrification inhibition—A novel strategy to regulate nitrification in agricultural systems. Adv. Agron..

[65] Di T., Afzal M.R., Yoshihashi T., Deshpande S., Zhu Y., Subbarao G.V. (2018). Further insights into underlying mechanisms for the release of biological nitrification inhibitors from sorghum roots. Plant Soil.

[66] Egenolf K., Verma S., Schöne J., Klaiber I., Arango J., Cadisch G., Neumann G., Rasche F. (2021). Rhizosphere pH and cation-anion balance determine the exudation of nitrification inhibitor 3-epi-brachialactone suggesting release via secondary transport. Physiol. Plant..

[67] Zhu Y., Zeng H., Shen Q., Ishikawa T., Subbarao G.V. (2012). Interplay among NH 4+ uptake, rhizosphere pH and plasma membrane H+-ATPase determine the release of BNIs in sorghum roots–possible mechanisms and underlying hypothesis. Plant Soil.

[68] Battey N.H., Blackbourn H.D. (1993). The control of exocytosis in plant cells. New Phytol..

[69] Subbarao G.V., Nakahara K., Ishikawa T., Yoshihashi T., Ito O., Ono H., Ohnishi-Kameyama M., Yoshida M., Kawano N., Berry W. (2008). Free fatty acids from the pasture grass Brachiaria humidicola and one of their methyl esters as inhibitors of nitrification. Plant Soil.

[70] Jung J., Yeom J., Kim J., Han J., Lim H.S., Park H., Hyun S., Park W. (2011). Change in gene abundance in the nitrogen biogeochemical cycle with temperature and nitrogen addition in Antarctic soils. Res. Microbiol..

[71] Hannula S.E., Heinen R., Huberty M., Steinauer K., De Long J.R., Jongen R., Bezemer T.M. (2021). Persistence of plant-mediated microbial soil legacy effects in soil and inside roots. Nat. Commun..

[72] Perkins L.B., Nowak R.S. (2013). Native and non-native grasses generate common types of plant–soil feedbacks by altering soil nutrients and microbial communities. Oikos.

[73] Rout M.E., Callaway R.M. (2012). Interactions between exotic invasive plants and soil microbes in the rhizosphere suggest that ‘everything is not everywhere’. Ann. Bot..

[74] Grove S., Parker I.M., Haubensak K.A. (2015). Persistence of a soil legacy following removal of a nitrogen-fixing invader. Biol. Invasions.

[75] Geddes P., Grancharova T., Kelly J.J., Treering D., Tuchman N.C. (2014). Effects of invasive Typha× glauca on wetland nutrient pools, denitrification, and bacterial communities are influenced by time since invasion. Aquat. Ecol..

[76] Stark J.M., Norton J.M. (2015). The invasive annual cheatgrass increases nitrogen availability in 24-year-old replicated field plots. Oecologia.

[77] Lee M.R., Flory S.L., Phillips R.P. (2012). Positive feedbacks to growth of an invasive grass through alteration of nitrogen cycling. Oecologia.

[78] Bansal S., Sheley R.L., Blank B., Vasquez E.A. (2014). Plant litter effects on soil nutrient availability and vegetation dynamics: Changes that occur when annual grasses invade shrub-steppe communities. Plant Ecol..

[79] Amatangelo K.L., Dukes J.S., Field C.B. (2008). Responses of a California annual grassland to litter manipulation. J. Veg. Sci..

[80] Sheley R., James J., Smith B., Vasquez E. (2010). Applying ecologically based invasive-plant management. Rangel. Ecol. Manag..

[81] Carey C.J., Blankinship J.C., Eviner V.T., Malmstrom C.M., Hart S.C. (2017). Invasive plants decrease microbial capacity to nitrify and denitrify compared to native California grassland communities. Biol. Invasions.

[82] Suding K.N., Gross K.L., Houseman G.R. (2004). Alternative states and positive feedbacks in restoration ecology. Trends Ecol. Evol..

[83] Hawkes C.V., Belnap J., D’Antonio C., Firestone M.K. (2006). Arbuscular mycorrhizal assemblages in native plant roots change in the presence of invasive exotic grasses. Plant Soil.

[84] Novoa A., González L., Moravcová L., Pyšek P. (2013). Constraints to native plant species establishment in coastal dune communities invaded by Carpobrotus edulis: Implications for restoration. Biol. Conserv..

[85] Torres N., Herrera I., Fajardo L., Bustamante R.O. (2021). Meta-analysis of the impact of plant invasions on soil microbial communities. BMC Ecol. Evol..

[86] Williams J.P., Gornish E.S., Barberán A. (2022). Effects of buffelgrass removal and nitrogen addition on soil microbial communities during an extreme drought in the Sonoran Desert. Restor. Ecol..

[87] Li K., Veen G., Ten Hooven F.C., Harvey J.A., van Der Putten W.H. (2023). Soil legacy effects of plants and drought on aboveground insects in native and range-expanding plant communities. Ecol. Lett..

[88] Xu Z., Guo X., Caplan S.J., Li M., Guo W. (2021). Novel plant-soil feedbacks drive adaption of invasive plants to soil legacies of native plants under nitrogen deposition. Plant Soil.

[89] Bais H.P., Weir T.L., Perry L.G., Gilroy S., Vivanco J.M. (2006). The role of root exudates in rhizosphere interactions with plants and other organisms. Annu. Rev. Plant Biol..

[90] De Long J.R., Fry E.L., Veen G., Kardol P. (2019). Why are plant–soil feedbacks so unpredictable, and what to do about it?. Funct. Ecol..

[91] Vinton M.A., Goergen E.M. (2006). Plant–soil feedbacks contribute to the persistence of Bromus inermis in tallgrass prairie. Ecosystems.

[92] Li W., Bi X., Zheng Y. (2023). Soil legacy effects on biomass allocation depend on native plant diversity in the invaded community. Sci. Prog..

[93] Perry L.G., Blumenthal D.M., Monaco T.A., Paschke M.W., Redente E.F. (2010). Immobilizing nitrogen to control plant invasion. Oecologia.

[94] Weidlich E.W., Flórido F.G., Sorrini T.B., Brancalion P.H. (2020). Controlling invasive plant species in ecological restoration: A global review. J. Appl. Ecol..

[95] Morghan K.R., Seastedt T. (1999). Effects of soil nitrogen reduction on nonnative plants in restored grasslands. Restor. Ecol..

[96] Cione N.K., Padgett P.E., Allen E.B. (2002). Restoration of a native shrubland impacted by exotic grasses, frequent fire, and nitrogen deposition in southern California. Restor. Ecol..

[97] Monaco T.A., Johnson D.A., Norton J.M., Jones T.A., Connors K.J., Norton J.B., Redinbaugh M.B. (2003). Contrasting responses of Intermountain West grasses to soil nitrogen. Rangel. Ecol. Manag. J. Range Manag. Arch..

[98] Huddleston R.T., Young T.P. (2005). Weed control and soil amendment effects on restoration plantings in an Oregon grassland. West. N. Am. Nat..

[99] Bleier J.S., Jackson R.D. (2007). Manipulating the quantity, quality, and manner of C addition to reduce soil inorganic N and increase C4: C3 grass biomass. Restor. Ecol..

[100] Eschen R., Mortimer S.R., Lawson C.S., Edwards A.R., Brook A.J., Igual J.M., Hedlund K., Schaffner U. (2007). Carbon addition alters vegetation composition on ex-arable fields. J. Appl. Ecol..

[101] Iannone III B.V., Galatowitsch S.M., Rosen C.J. (2008). Evaluation of resource-limiting strategies intended to prevent Phalaris arundinacea (*reed canarygrass*) invasions in restored sedge meadows. Ecoscience.

[102] Uddin M.N., Robinson R.W., Asaeda T. (2020). Nitrogen immobilization may reduce invasibility of nutrient enriched plant community invaded by Phragmites australis. Sci. Rep..

[103] James J., Drenovsky R., Monaco T., Rinella M. (2011). Managing soil nitrogen to restore annual grass-infested plant communities: Effective strategy or incomplete framework?. Ecol. Appl..

[104] Haerdtle W., Niemeyer M., Niemeyer T., Assmann T., Fottner S. (2006). Can management compensate for atmospheric nutrient deposition in heathland ecosystems?. J. Appl. Ecol..

[105] Kardol P., Van der Wal A., Bezemer T.M., de Boer W., Duyts H., Holtkamp R., Van der Putten W.H. (2008). Restoration of species-rich grasslands on ex-arable land: Seed addition outweighs soil fertility reduction. Biol. Conserv..

[106] EA H. (2005). Nitrogen deposition onto the United States and Western Europe: Synthesis of observation and models. Ecol. Appl..

[107] Prober S.M., Lunt I.D. (2009). Restoration of Themeda australis swards suppresses soil nitrate and enhances ecological resistance to invasion by exotic annuals. Biol. Invasions.

[108] Chen W.B., Chen B.M., Liao H.X., Su J.Q., Peng S.L. (2020). Leaf leachates have the potential to influence soil nitrification via changes in ammonia-oxidizing archaea and bacteria populations. Eur. J. Soil Sci..

[109] Abalos D., van Groenigen J.W., Philippot L., Lubbers I.M., De Deyn G.B. (2019). Plant trait-based approaches to improve nitrogen cycling in agroecosystems. J. Appl. Ecol..

